# The Treatment of Ulcers in Charity Hospital, New York

**Published:** 1873-07

**Authors:** 


### Miscellaneous.

:o:

### The Treatment of Ulcers in Charity Hospital, N. Y.

We were present on one of the days which may be called a "field day" in the ulcer ward.

A general principle of treatment adopted in every case, with a single exception, that day, was that of strapping with adhesive plaster and using a firm roller bandage. Each ulcer was snugly strapped with either transverse or basket strapping, and the limb firmly bandaged.

The dressing for the ulcers was varied according to the requirements of each case, certain appearances indicating the use of certain applicatons. Some experience is requisite to recognize the exact condition requiring the use of a certain remedy which, if properly applied—applied in strict conformity to the indication—renders the general treatment much more satisfactory.

Examples of specific ulcers are somewhat numerous here. One was dressed with iodoform and glycerine (sat. sol.) before applying the straps. It requied a little stimulating.

For all these cases of gangrenous and sloughing ulcers either a saturated solution of carbolic acid is used, or a solution of bromine, grs. xvi. to the ounce of water.

Traumatic ulcers generally receive the extra attention of scraping epidermal scales from the leg with a pocket-knife and spreading the surface of the ulcer. Since this has been practised, the ulcer has healed much more rapidly than before, with no different treatment otherwise. Over these scales was placed a dressing of balsam of Peru, and over this the straps and bandage as usual. Covering the surface of the ulcer with a coating of balsam of Peru is the ordinary method of dressing all ulcers that require no special application, and it is quite generally used in connection with the special application. For example, balsam of Peru and iodoform (in powder) act together better than either alone. One is soothing and the other stimulating, and the joint benefit received from the combination is much greater than that received from either alone. When ulcers are covered with exuberant granulation, the solid stick of nitrate of siver is applied, and over this the ordinary dressing.

Ulcers with thickened edges are reduced by freely cutting through the indurated mass in transverse incisions throughout the entire circumference of the ulcer.

The treatment of varicose ulcers forms no exception to the general rule.

A man who had both legs covered with ulcers, from half an inch to an inch in diameter, resulting from hard service and low salt diet at sea, received the iodoform dressing, the ulcers being neither strapped nor bandaged.

An old man in bed with an ulcer involving about two-thirds of the circumference of his leg, which had been in an extremely sluggish condition, now exhibits a lively growth of healthy looking granulations all over its surface, which had been established within three or four days by the use of the following :

| | | |
|---|---|---|
| ℞ | Flour | ℥ iv. |
| | Gum tragacanth | ℥ ss. |
| | Gum arabic | ℥ j. |
| | Egg. No | i. |
| | Chalk | ʒ iij. |

Make a paste by adding boiling water, and while fresh place it on the ulcer with a brush, four times a day. When the material sours it must be changed.

One great advantage derived from this general plan of treatment by strapping and bandaging is, that the patient can go about without detriment to the ulcer.—*Medical Record.*

